# Stress Overshoot Analysis in Flow Start-Up Tests: Aging Time Fitting of the Different Gel-Based Drilling Fluids

**DOI:** 10.3390/gels11020127

**Published:** 2025-02-10

**Authors:** Luis H. Quitian-Ardila, Raquel S. Schimicoscki, Yamid J. Garcia-Blanco, Eduardo M. Germer, Vladimir Ballesteros-Ballesteros, Oriana Palma Calabokis, Admilson T. Franco

**Affiliations:** 1 Research Center for Rheology and Non-Newtonian Fluids–CERNN, Federal University of Technology–Paraná, Curitiba 81280-340, PR, Brazil; schimicoscki@yahoo.com.br (R.S.S.); yamidblanco@alunos.utfpr.edu.br (Y.J.G.-B.); eduardomg@utfpr.edu.br (E.M.G.); admilson@utfpr.edu.br (A.T.F.); 2 Faculty of Engineering and Basic Sciences, Fundación Universitaria Los Libertadores, Bogotá 110231, Colombia; vladimir.ballesteros@libertadores.edu.co (V.B.-B.); opalmac@libertadores.edu.co (O.P.C.)

**Keywords:** flow start-up, gels, gel-like behavior, stress overshoot, yield stress

## Abstract

Drilling fluids are essential for maintaining cutting suspension during drilling, exhibiting gel-like behavior at rest and liquid-like behavior under shearing. These fluids display shear-thinning behavior, yield stress, and thixotropy. This study investigates the impact of aging time on stress overshoot and the deformation required to disrupt the gelled structure of water-based and synthetic-based drilling fluids. Flow start-up tests were conducted using a rotational rheometer at 25 °C and atmospheric pressure. The results show that aging time significantly affects both stress overshoot and the shear strain needed to disrupt the gelled structure. Longer aging times reduce the strain required to break the structure, indicating a correlation between aging time and stress overshoot. The fitted model strongly correlates with the experimental data, providing valuable insights for the planning and simulation of offshore drilling wells, primarily in well stability.

## 1. Introduction

The petroleum industry relies heavily on drilling fluids for the efficient and successful execution of the drilling process [1]. These fluids play a crucial role in various aspects; one of the principal functions is maintaining the suspension of cuttings during process stoppages, preventing bit obstructions, and ensuring wellbore stability [2,3]. Drilling fluids are considered soft matter, characterized by their complex structure and flexibility [4]. Some drilling fluids may consist of biopolymers, oils, solid mixtures, etc., but their main characteristic is viscoplastic behavior [5] with low elasticity, often referred to as gels. A gel is a system consisting of a structure-forming component and an absorbed liquid, typically a low-viscosity solvent, primarily composed of polymeric fluid compounds, concentrated emulsions, foams, and other colloids [6].

Drilling fluids are categorized as materials capable of exhibiting two distinct modes of behavior when subjected to mechanical loading. These materials undergo irreversible deformations and, in some cases, exhibit yield stress [7], which is explained by the strength of their internal structure. Viscoelastic-plastic fluids display three well-defined zones during the application of a constant deformation [8]: solid-like, or more specifically as gels; gel-like, representing the transition from solid-like to liquid-like; and finally, a fully destructured zone behaving as a liquid state [4]. This distinction between the two types of rheological behavior relates to the classification of gels into chemical and physical gels. Chemical gels are characterized by permanent covalent bonds, whereas physical gels [9], such as colloidal gels [10], are defined by temporary and weak interactions.

Gels are chemically cross-linked substances that form complex structures through covalent or ionic bonds [11]. The formation of this polymer network, maintained by chemical bridges or physical interactions, ensures that the fluid retains essential properties, such as particle suspension and appropriate viscosity [12]. Control over gelation is achieved by adjusting the polymer concentration, selecting the type of crosslinking agent, and optimizing the temperature and pressure conditions within the drilling environment [13]. Gel-like behavior in drilling fluids arises from interactions between particles and additives, such as polymers, which form a reversible three-dimensional network [4,14]. This network can be activated by variations in temperature and pressure or by the addition of gelling agents [13]. The ability of drilling fluids to exhibit gel-like behavior at rest and rapidly transition into a liquid-like state under shear stress is crucial for efficient drilling operations. Physically, a gel can be defined as a soft non-fluid elastic substance with a fully structured permanent network. When an external force is applied, it undergoes reversible viscoelastic deformations [15]. However, upon reaching a critical stress threshold, the structure breaks down, and the gel flows like a viscous fluid [4]. During flow start-up, these fluids often require pressures significantly higher than normal to disrupt their gelled structure [16]. These pressures can exceed the formation’s fracture threshold, posing a risk of damaging the wellbore walls [13,17]. Therefore, understanding their rheological behavior, particularly the stress overshoot phenomenon, becomes critically important [18,19].

Drilling fluids have a complex composition that gives rise to unique non-Newtonian rheological behavior, specifically shear-thinning, characterized by properties such as yield stress and thixotropy [17,20]. Understanding these rheological properties is essential, as they directly influence the performance and efficiency of drilling operations. The stress overshoot phenomenon, which refers to the transient increase in stress experienced by the fluid when subjected to rapid deformation, reaches a maximum value before returning to a steady-state value, providing valuable insights into the behavior of these drilling fluids during critical operational stages [21,22]. Moreover, aging time is a crucial parameter affecting the rheological properties of drilling fluids [23]. Aging refers to the time interval between the preparation of the drilling fluid and its usage in the field [18].

The stress overshoot phenomenon, arising from the interplay between material elasticity and thixotropy [24], has been extensively studied [25,26]. This behavior is often observed in experiments where shear rates are precisely controlled and monitored using rotational rheometers. Notable studies by Fernandes et al. [27] support these findings. Researchers have developed structural kinetic models incorporating a structure parameter to quantify material structuring, offering deeper insights into this phenomenon [28]. This parameter varies with shear rate, ranging from zero—indicating a completely unstructured material disrupted by shear—to a finite value, representing a fully structured material at rest. These metrics provide a detailed understanding of reversible changes in material behavior under different shear and rest conditions [29]. In the industry, this phenomenon is motivated by the planning of drilling activities at different stages of drilling, primarily in the sizing of equipment and the arrangement of facilities for the use of newly formulated fluids. Therefore, the analysis and understanding of this phenomenon are crucial for operational safety and drilling efficiency. In their quest to fully understand the rheological behavior of elastoviscoplastic thixotropic materials, some researchers have focused on fitting structural kinetic models to experimental data [28]. Models have demonstrated strong agreement with rheometric data in steady states and during transient phases [30]. Similarly, Negrão et al. [18] applied the model by Dullaert and Mewis [21] to rheometric data for an oil-based drilling fluid. Using a shear rate-controlled test to simulate the initiation of the gelled material, they found that the model could accurately predict flow start-up stress overshoot with slight modifications. However, the model struggled to predict the overshoot time accurately, which was consistently shorter than the computed values [31].

The oil and gas industry demands simple models to aid in well planning, particularly under high-pressure high-temperature conditions [25,32,33]. Existing studies attempt to predict elastic and viscous forces during flow restart—a complex task involving many parameters [7,17,32,33], leading to higher computational costs. Since flow restart and stress overshoot values are critical in industrial operations, a simple model capable of evaluating various conditions for different fluids is highly advantageous. Furthermore, the time-dependent aging behavior of drilling fluids creates additional complexities, as more sophisticated models often delay the identification of stress overshoot values.

This study focuses on the influence of aging time on stress overshoot and the deformation required to disrupt the gelled structure. Understanding the effect of aging time is essential, as it directly impacts the performance and stability of drilling fluids during operations. To address these challenges, a simplified equation with fewer parameters was used to fit experimental data from flow start-up tests, facilitating its application in oilfield operations. Unlike previous approaches, this study integrates a time-dependent function into the Herschel–Bulkley model, enabling predictions of shear stress as a function of aging time and shear rate. The findings are expected to reveal a correlation between aging time and stress overshoot, suggesting that longer aging times may reduce the deformation required to disrupt the gelled structure, offering practical insights for the industry.

## 2. Results and Discussion

### 2.1. Steady-State Flow Curve

**Figure 1:**
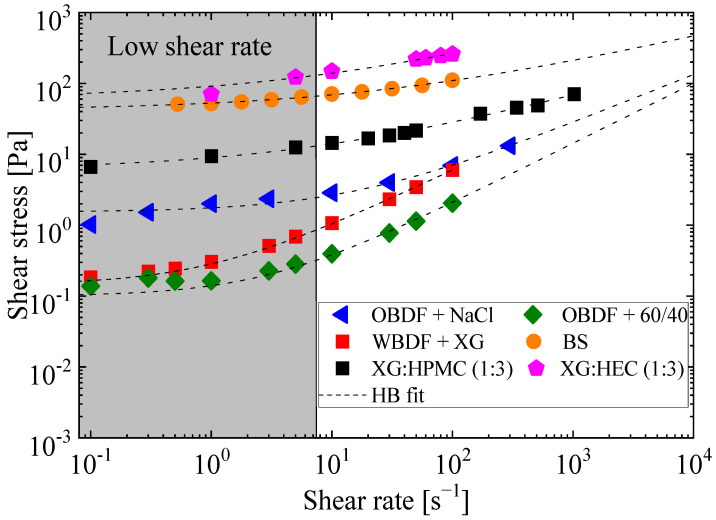
Steady-state flow curves for the various analyzed drilling fluids. The measurements were carried out in triplicate and fitted by the Herschel–Bulkley model.

Steady-state flow curves were conducted at 25 °C at atmospheric pressure for the different drilling fluids analyzed. Figure 1 shows the shear stress as a function of the shear rate and the Herschel–Bulkley fitting model for each fluid. As illustrated in Figure 1 on a logarithmic scale, all six fluids demonstrate shear-thinning behavior. This behavior is characterized by an increase in shear rate with an increase in shear stress. When the fluids exhibit yield stress, regions of constant values at low shear rates can be observed, followed by a nonlinear behavior. To better understand these rheological behaviors, it is essential to consider the influence of the microstructure of the drilling fluids. These fluids, known for their non-Newtonian properties, exhibit complex behaviors directly influenced by their microstructural interactions. Specifically, the gels within these fluids possess elasto-viscoplastic and thixotropic properties, resulting from interactions among solid particles, polymers, and dispersing media. These micromechanisms are critical in forming and breaking structural networks, directly impacting macroscopic behaviors during operations such as flow restart [33].

Among the analyzed fluids, the bentonite suspension (BS) demonstrates the most pronounced behavior, exhibiting high dynamic yield stress compared to other fluids, including the oil-based drilling fluid (OBDF) with NaCl, the xanthan gum–Hydroxypropyl Methylcellulose (XG-HPMC) mixture, and the xanthan gum–Hydroxyethyl Cellulose (XG-HEC) mixture. In contrast, the water-based drilling fluid (WBDF) with xanthan gum (XG) and the OBDF with a 60/40 oil/water ratio show lower yield stress. The rheological characteristics of these drilling fluids vary significantly, as evidenced by differences in dynamic yield stress and viscosity, resulting in a wide range of fluid behaviors. The bentonite suspension spans from simpler viscoelastic fluids with minimal yield stress to viscoplastic and elastoviscoplastic fluids characterized by yield stress and thixotropic behavior. Understanding these variations is crucial for optimizing the performance of drilling fluids in critical operations, such as flow restart, and maintaining well stability under varying stress conditions.

The steady-state flow curves were fitted to the Herschel–Bulkley model, as shown in Figure 1, allowing for an analysis of the varying behaviors that drilling fluids exhibit with changes in shear rate. The fitting parameters for the four flow curves are detailed in Table 1, offering insights into the yield stress, consistency coefficient (m), flow behavior index (n), and correlation coefficient ($R^{2}$), which assesses how well the curves align with the experimental data.

Table 1 presents a comprehensive overview of the drilling fluid’s rheological properties. Notably, the bentonite suspension displayed a yield stress of approximately 42 Pa and a behavior index of 0.401. A substantial shear-thinning behavior within this fluid is signified by these values collectively. The fluid’s apparent viscosity decreases as the shear rate increases, implying this behavior, which is a critical characteristic in drilling operations where efficient circulation and suspension of cuttings are paramount.

In contrast, the remaining drilling fluids displayed significantly lower yield stresses of 1.53, 0.14, and 0.10 Pa, respectively. Considering the last two, water-based drilling fluids with xanthan gum and the oleophilic drilling fluid with a 60/40 oil-to-water ratio, these values are notably lower and almost negligible. Such low-yield stresses indicate their relative ease in responding to applied stresses and flowing, making them suitable choices for specific drilling conditions. The microstructures of each fluid analyzed here exhibit different interactions, but it can be observed that in the case of the bentonite suspension and the biopolymer blends, stronger connections and more interconnected microstructures were present, which may have resulted in higher yield stress values.

Moreover, the behavior indices measuring fluid consistency and flow behavior were more significant than 0.6 for most fluids studied, indicating a behavior that tends toward Newtonian fluids, which exhibit consistent viscosity regardless of the shear rate. Understanding these rheological characteristics is pivotal in the oil industry, as it informs the selection of drilling fluids best suited for various operational scenarios and wellbore conditions, in addition to understanding that fluids with gel-like behavior exhibit similar structuring characteristics. Understanding the behavior of different fluids in this stable state provides a clearer insight into selecting the shear rates explored during transient states and the parameters that can influence the flow start-up for these fluids.

### 2.2. Analysis Stress Overshoots—Yielding

The rheometric data obtained from the tests were analyzed to investigate stress overshoot (gel strength) phenomena and the impact of aging time on the fluid’s behavior. The stress overshoot measured in constant shear-rate experiments provides estimates of characteristic stresses similar to the static yield stress obtained in other experiments.

The evolution of shear stress over time offers intriguing insights into material behavior. Initially, as shear stress increases, this rise is primarily due to the material’s inherent elasticity, as observed in previous studies [8,17]. The material’s response to applied forces is illustrated in this phase, where elasticity plays a dominant role. However, a notable phenomenon emerges as time progresses: the stress overshoot. The transition from a gel-like consistency to a more liquid state [27] is marked by this overshoot, a complex process that is influenced by the material’s thixotropic nature, where the apparent viscosity changes over time [34], and its viscoelastic properties.

**Figure 2:**
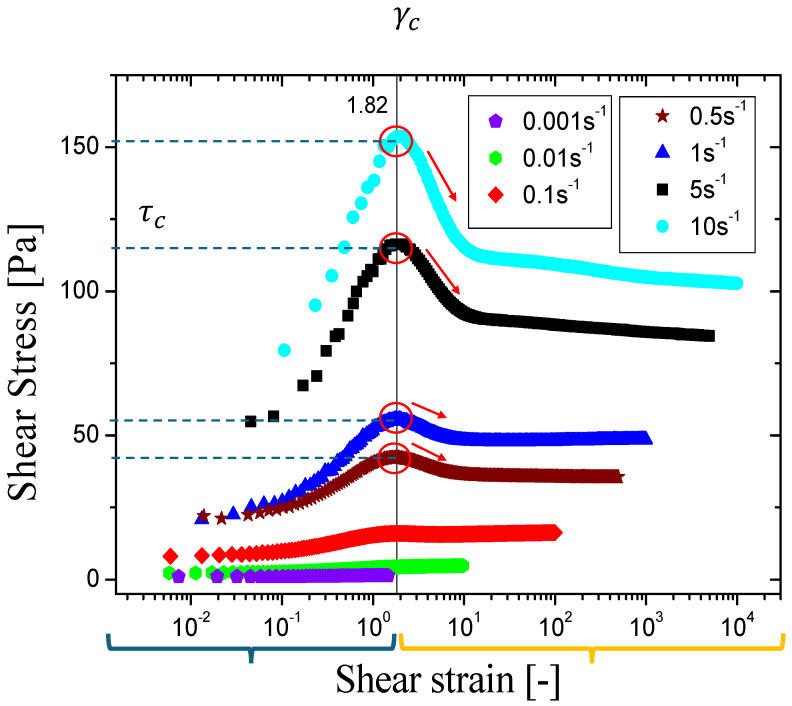
Typical results of the flow start-up test in viscoelastoplastic-thixotropic fluids displaying shear stress as a function of shear strain. The red circles indicate the shear stress peak, known as stress overshoot. The red arrows are pointing out that after reaching the maximum point, the microstructure is irreversibly broken, resulting in the fluid’s viscous behavior.

In essence, the evolution of shear stress over time is a valuable indicator of material behavior, revealing insights into elasticity and the transition from a gel-like to a more liquid state, driven by thixotropy and viscoelasticity [33]. Understanding this is crucial for applications with key material flow and deformation characteristics. We examine the material in the liquid-like regime to analyze the transition from solid-like to liquid-like behavior. After allowing the material to rest for a specified time interval, a constant shear rate is applied and maintained until a steady state is achieved, as shown in Figure 2.

This behavior was observed for biopolymer mixtures of XG and HEC, with an aging time of 600 s, and each shear rate was maintained for 1000 s. Upon analyzing the shear stress, we observe that the material undergoes initial deformation, followed by a sharp drop in shear stress. The peak in shear stress, known as stress overshoot ($\tau_{c})$, is a critical indicator of the transition from elastic to viscous behavior (see Figure 2). At this same point, the critical strain ($\gamma_{c}$) is identifiable, indicating the moment of transition from a solid-like to a liquid-like regime. The behavior of shear stress as a function of shear strain, shown in Figure 2, is characteristic of materials with viscoelastoplastic properties.

In the majority of materials, the increase in stress overshoot with aging time can be explained by the evolution of the microstructure. During aging, the density of interactions between particles and polymers increases, resulting in stiffer networks that require greater stress to break. This observation aligns with studies correlating the macroscopic behavior of gels and non-Newtonian fluids with microscopic processes, such as network densification or the strengthening of intermolecular bridges [33].

This comprehensive analysis provides valuable insights into the rheological behavior of these drilling fluids under different conditions, shedding light on their performance in real-world drilling operations.

In our analysis, we took the experimental data and subjected it to a comprehensive fitting process. Utilizing a nonlinear fitting model, specifically employing the least squares method, was involved to ensure the best fit. The Herschel–Bulkley model [Equation (1)] was incorporated to describe the rheological behavior of the materials under study, coupled with a function that accounted for the influence of rest time, as shown in Equation (2). The critical parameter estimation task was executed using the Levenberg–Marquardt method, a robust optimization technique. The meticulous approach allowed us to accurately capture and model the complex rheological properties of the materials, facilitating a deeper understanding of their behavior.

(1) $$\tau =\tau_{0}+m{{\dot{\gamma }}_{p}}^{n};[Pa]$$

(2) $$\tau_{Overshoot}=\left(\tau_{0}+m{{\dot{\gamma }}_{p}}^{n}\right)f\left(t_{r}\right);\;[\mathrm{Pa}]\;f\left(t_{r}=0\right)=1$$

where $\tau_{Overshoot}$ [Pa] is defined by $\tau_{0}$ [Pa], which represents the yield stress, m [Pa.s^n^] stands for the consistency coefficient, n [-] denotes the flow behavior index, ${\dot{\gamma }}_{p}$ [s^−1^] signifies the peak shear stain, $f\left(t_{r}\right)$ [-] is a function that accounts for the variation in sample resting time, and $t_{r}$ [s] represents aging time. Therefore, when this function equals zero, its value becomes one, signifying that the fluid’s behavior proportionally adheres to the Herschel–Bulkley model.

(3) $$f\left(t_{r}\right)=\frac{A+t_{r}}{A+Bt_{r}}+Ct_{r};\;[-]$$

In this equation, A, B, and C are fitting coefficients that precisely explain how a fluid’s time at rest impacts its response to subsequent stresses or shearing. The term ($A+t_{r}$) in the numerator represents a gradual increase in the property or behavior of the fluid as the resting time increases, while the denominator ($A+Bt_{r}$) modulates this influence. Additionally, the additional term $Ct_{r}$ adds a linear contribution to the value of $f\left(t_{r}\right).$ In summary, this equation is valid for characterizing and predicting how fluid behaves after different resting periods, which is crucial in applications involving the manipulation of viscous or rheologically complex fluids.

**Figure 3:**
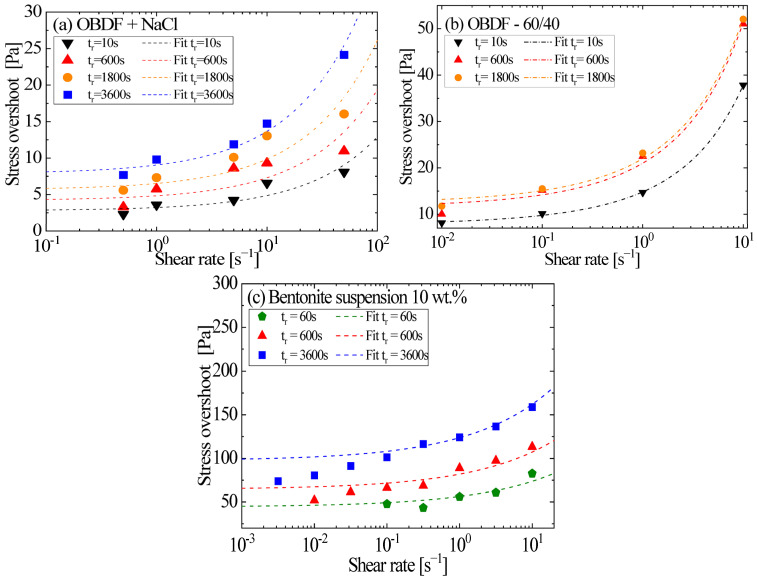
Comparison between the experimental results and the fits for the (**a**) olefin-based with NaCl, (**b**) olefin-based 60/40 oil/water, and (**c**) bentonite suspensions.

The flow start-up test was conducted for all drilling fluids described in Section 4, utilizing various shear rates and aging times. Experimental data from stress–strain curves obtained through rheological tests were processed to determine the maximum stress values (stress overshoots). The values were plotted for each drilling fluid as a function of the shear rate, as depicted in Figure 3. Figure 3a presents the experimental data for the olefin-based drilling fluid with NaCl. Figure 3b displays the olefin-based drilling fluid with a 60/40 oil-to-water ratio and Figure 3c shows the values of the bentonite suspension.

Figure 3 presents the stress overshoot responses of the drilling fluids with different aging times and shear rates. A rheometer was used to obtain these responses, which were fitted using the correlations in Equations (2) and (3) (dotted lines). The stress overshoot increases in varying proportions with aging time, depending on the material’s microstructure and the concentration of its primary components. The behavior observed in Figure 3a for the olefin-based drilling fluid with NaCl exhibited nearly proportional increases. However, the drilling fluid with a 60/40 oil-to-water ratio, as shown in Figure 3b, displayed an insignificant increase when left to rest for 600 s. Even with an extended aging time of 1800 s, only a slight increase occurred, differing from the NaCl-based drilling fluid. Typically, as observed, the stress evolution during start-up flow of a gelled fluid can be explained in terms of the combined elastic and viscous responses [32]. Initially, the elastic structure resists deformation, leading to a stress peak (overshoot). The strength of this peak depends on the density and rigidity of the microstructure, as seen in the bentonite suspension (Figure 3c), which exhibits higher yield stress and viscosity values. The bentonite suspension’s microstructure is dominated by strong particle–particle interactions and a high solid concentration, which resist shear deformation, resulting in a pronounced rise in stress overshoot with extended aging times.

We can observe that the behavior of stress overshoot values coincides with the results presented in the literature [17], where increases in stress overshoot values are observed with an increase in shear rate, indicating that the measurements from the start-up flow tests were accurately obtained. As we observed, the elastic stress increases with deformation, leading to higher peak values characterized by a yield point of the fluid, stabilizing to steady-state values. Additionally, it has been demonstrated that increasing the aging time for time-dependent fluids results in higher stress overshoot values [35].

**Figure 4:**
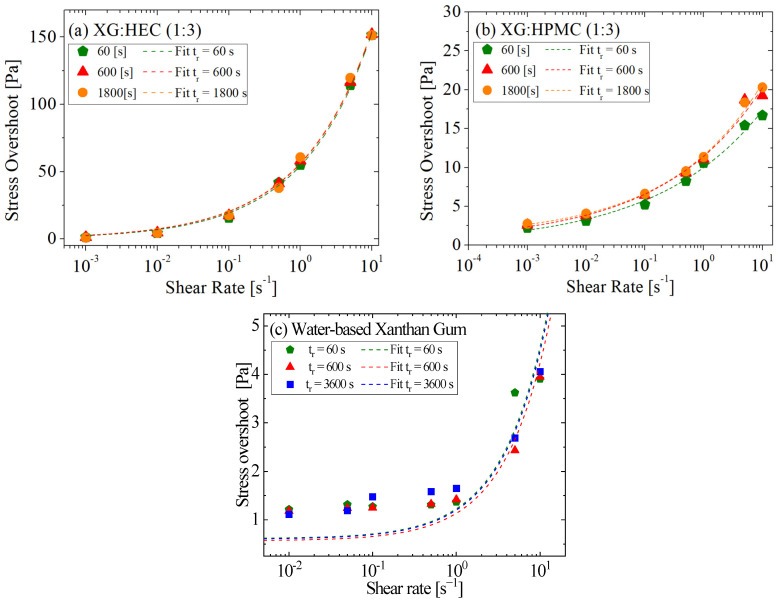
Comparison between the experimental results and the fits for (**a**) XG—HEC and (**b**) XG—HPMC suspensions and (**c**) water-based suspensions with xanthan gum.

For the biopolymer mixtures of XG and HEC, shown in Figure 4a, the behavior is independent of aging time. Even after extended resting periods, the fluid does not exhibit significant changes in stress overshoot values. This can be explained by the nature of the microstructure formed in these fluids, where the polymer network does not undergo significant strengthening or reorganization over time. Similarly, the XG and HPMC biopolymer mixture, shown in Figure 4b, displays comparable behavior, with no significant modifications in the stress overshoot peak. As observed for some drilling fluids, the increase in stress overshoot did not correlate with aging time, indicating that these fluids exhibit the elastic-to-viscous transition [35,36] but do not undergo significant material restructuring over time. The elastic response, responsible for the stress overshoot, is determined by the fluid’s ability to form and maintain a robust network during rest.

Figure 3c and Figure 4c show a similar trend for the water-based fluids. In the case of the bentonite suspension, a substantial increase in stress overshoot is evident with an extended aging time. This behavior is due to the high concentration of solid particles and their ability to form a stable interconnected network. Conversely, for the drilling fluid with xanthan gum, the increase in aging time had a nearly negligible impact. This can be attributed to the limited ability of the xanthan gum-based microstructure to densify or strengthen during rest, leading to a lower dependence on aging time.

The distinct stress overshoots are illustrated across various aging times. As depicted in Figure 3 and Figure 4, it becomes evident that a correlation exists between the escalation of stress overshoot and both rest time and shear rate. Enhanced stability is noticeable with prolonged rest times for the olefin-based drilling fluid with a 60/40 oil-to-water ratio (Figure 4c). Increased stress overshooting is observed for both low and high shear rates, accompanying this trend. In contrast, the bentonite suspension (Figure 3c) exhibits higher yield stress and viscosity values and does not display sedimentation. Consequently, there is a pronounced rise in stress overshoot as the resting time extends, reflecting the strengthening and densification of its microstructure.

A noteworthy observation was made in the water-based drilling fluid with xanthan gum (Figure 4c); the stress overshoot diminished as the resting time increased, particularly at lower shear rates. However, this effect was not replicated at higher shear rates. This lack of agreement may be related to the margin of error of the equipment or to minor structural breakdowns occurring over extended rest periods. The decreasing stability of the fluid over prolonged periods could potentially be linked to these factors.

It is worth noting that the fitting process yielded effective results for both the bentonite suspension and the olefin-based drilling fluids. Nevertheless, the outcomes for the water-based drilling fluid with xanthan gum did not exhibit a straightforwardly proportional relationship with resting time, further highlighting the differences in microstructural behaviors across these fluids.

For all fluids, the simple model adapted well to their behavior, particularly regarding the increase in stress overshoot. The rheological measurements were analyzed through ANOVA analysis to identify their statistical significance. All fits were statistically significant, as indicated by their *p*-values (<0.001), thus ensuring the combined expanded uncertainty of the stress overshoot and shear rate measurements at the 95% confidence level. All the fits were adequately representative, with a correlation coefficient exceeding 0.98. Sedimentation may explain this phenomenon, as observed in [17] when the fluid rests for 600 s.

The practical implications of these discoveries extend significantly as they improve the efficiency, safety, and cost-effectiveness of drilling operations within the oil and gas sector. In summary, this scientific article delves deep into the complex rheological characteristics of drilling fluids, shedding light on the influence of stress overshoot and aging time on the disruption of the gelled structure. The knowledge acquired from this research holds substantial promise for driving progress in drilling fluid technology and streamlining drilling activities, especially in offshore environments. The successful fitting of the models to experimental data and the established correlations establishes a robust foundation for further exploring and applying these findings in practical drilling operations.

The increase in stress overshoot values with resting time can be attributed to microstructural reorganization, where there is an increase in the density of temporary bonds or the strength of existing interactions. This hypothesis is supported by previous studies that correlate the evolution of microstructure with changes in rheological properties, such as elasticity and thixotropy [33]. An essential aspect is understanding how the microstructure of the samples is correlated with the forces determined by the stress overshoot. To deepen this analysis, future studies could integrate advanced experimental techniques, such as confocal microscopy or small-angle X-ray scattering. These techniques would enable the visualization of structural evolution during flow start-up tests, providing a stronger connection between microscopic phenomena and macroscopic behavior. This approach would further enhance the understanding of the relationships between the microstructural organization and the macroscopic rheological response, offering valuable insights for optimizing the performance of gel-based drilling fluids in field operations.

## 3. Conclusions

The results of this study demonstrate that the fitted models, based on the Herschel–Bulkley equation modified by the selected rest time function, show an excellent fit to the experimental data obtained from flow start-up tests on various drilling fluids. The correlations identified between stress overshoot, rest time, and shear rate provide valuable insights into the rheological behavior of these fluids during critical operational stages. The accuracy of these models suggests their potential application in flow start-up tests for other fluids used during well drilling or production. However, it is crucial to validate their predictive capability across a broader range of fluids through additional testing, analyzing other parameters such as thixotropy and the addition of particulate material. The implementation of the model will greatly assist in the planning and simulation processes of flow start-up in oil wells during various operational shutdowns, preventing issues ranging from potential well instabilities to equipment sizing. The discrepancy observed in the behavior of the water-based drilling fluid may be attributed to a delay in the rheometer’s application of the shear rate, which could have influenced the stress overshoot values. Therefore, when dealing with fluids that exhibit rapid changes in rheological properties, careful consideration of the experimental setup and measurement devices is essential to ensure accurate data acquisition.

Although the results and correlations obtained may be specific to the fluids tested or those with similar rheological characteristics, the findings are valuable for enhancing the planning and simulation of drilling operations in offshore fields. The ability to predict stress overshoot and understand the impact of aging time on the disruption of the gelled structure of drilling fluids can contribute to improving the efficiency, safety, and cost-effectiveness of drilling processes in the oil and gas industry. Future research should prioritize validating these models across a broader spectrum of fluids and refining experimental procedures to address potential limitations.

## 4. Materials and Methods

Different drilling fluids were formulated to analyze the effect of aging time on stress overshoot. The study examined fluids such as a bentonite suspension (BS), an oil-based drilling fluid (OBDF) containing NaCl, the xanthan gum–Hydroxypropyl Methylcellulose (XG-HPMC) mixture, and the xanthan gum–Hydroxyethyl Cellulose (XG-HEC) mixture, a water-based drilling fluid (WBDF) with xanthan gum (XG), and an OBDF with a 60/40 oil/water ratio and a barite concentration of 11.5 lb./bbl. The subsequent Section 4.1 provides details on each fluid formulated and kindly provided by Petrobras, followed by Section 4.2, which outlines the methodology used for the rheological measurements.

### 4.1. Drilling Fluids

The rheological analysis of flow restart was performed using different drilling fluids employed in the main stages of drilling. The bentonite suspension, the xanthan gum–Hydroxypropyl Methylcellulose (XG-HPMC) mixture, and the xanthan gum–Hydroxyethyl Cellulose (XG-HEC) mixture were tested using an Anton Paar MCR 702TD (Anton Paar Co., Graz, Austria) rotational rheometer with a grooved parallel plate geometry. The suspension was prepared by manually mixing the powder with distilled water in a small bottle and left to hydrate and swell over the course of one week. Antifoaming and antifungal agents were then added. For the olefin-based drilling fluid, measurements were conducted on an Anton Paar MCR 702TD rheometer with sandblasted coaxial cylinders. An olefin-based composition mixed with a sodium chloride brine composed this drilling fluid.

On the other hand, the water-based drilling fluid, formulated with 0.25 wt.% xanthan gum, was evaluated using a TA-DHR 3 (TA Instruments, New Castle, DE, USA) rotational stress-controlled rheometer with coaxial cylinders featuring roughened surfaces. The fluid, containing NaCl brine, high-performance starch, and other additives, was analyzed for its rheological properties. Finally, a 60/40 oil-to-water ratio of olefin-based drilling fluid was tested on a Haake Mars III (Haake Co., Vreden, Germany) rheometer using grooved parallel plates. Fluid preparation included the addition of dispersed Barytine at a concentration of 11.5 pounds per gallon (ppg) to increase its density. The pre-test process for this fluid was consistent with that of the other olefin-based sample, ensuring an even distribution of Barytine in the test sample for accurate rheological measurements.

Before each measurement, the fluid was homogenized for 10 min using a Hamilton Beach HMD200 (Hamilton Beach Brands Inc., Glen Allen, VA, USA) shaker. The sample was then carefully loaded into each rheometer to minimize air entrapment and ensure measurement precision.

### 4.2. Rheological Measurements

For the rheological characterization of different drilling fluids, measurements were performed using rheometers with specified geometries, as mentioned in Section 4.1. Rheological tests of steady-state flow curves were conducted to characterize the various drilling fluids by imposing different shear rates (10^−1^ to 10^3^) for 1000 s. A 1% variability in shear stress values was used to determine when a steady state was achieved. Temperature control was maintained using the rheometer’s Peltier system and a thermal bath to keep the samples at 25 °C and atmospheric pressure. Before testing, the fluids were pre-sheared at 300 s^−1^ for 100 s, creating a state independent of the sample’s shear history and standardizing the fluid. Subsequently, different shear rates in a decreasing form were applied. The tests were performed in triplicate to ensure the measurements’ repeatability, homogeneity, and reliability.

Subsequently, flow restart tests were conducted to identify the stress overshoot values during the transition from solid to liquid regime. Shear rate-controlled experiments were performed to replicate the conditions of drilling fluid flow start-up [8,17,27,37]. Various shear rate values were imposed using a linear function, incrementing from zero to a final shear rate (0.003, 0.01, 0.02, 0.05, 0.1, 0.2, 0.5, 1, 2, 5, 10, and 50 s^−1^). During the experiment, the sample was pre-sheared at 300 s^−1^ for 100 s and then allowed to rest for specific durations of 10, 60, 600, 1800, or 3600 s, following the American Petroleum Institute [32,38] guidelines.

## Figures and Tables

**Table 1:** Herschel–Bulkley model fitting parameters for analyzed drilling fluids. Dynamic yield stress (τ_0_), consistency coefficient (m), flow behavior index (n), and corresponding correlation coefficients (*R*^2^).

Fluids	Dynamic Yield Stress $\tau_{0}$ (Pa)	Consistency Coefficient $m\;(Pa.s^{n})$	Flow Behavior Index n (-)	$R^{2}$	MSE
BS	42.182	10.722	0.401	0.998	0.541
OBDF + NaCl	1.531	0.234	0.691	0.993	0.847
WBDF + XG	0.142	0.140	0.809	0.999	1.548
OBDF60/40	0.104	0.045	0.851	0.997	0.501
XG:HPMC (1:3)	6.278	2.757	0.452	0.992	0.255
XG:HEC (1:3)	61.581	29.821	0.417	0.994	0.014

## Data Availability

The raw data supporting the conclusions of this article will be made available by the authors upon request.
